# Comparison of liver iron concentration calculated from R2* at 1.5 T and 3 T

**DOI:** 10.1007/s00261-022-03762-4

**Published:** 2022-12-15

**Authors:** Elisabeth Pickles, Shravan Kumar, Michael Brady, Alison Telford, Michael Pavlides, Daniel Bulte

**Affiliations:** 1 Institute of Biomedical Engineering, Department of Engineering Science, University of Oxford, Old Road Campus Research Building, Headington, Oxford, OX3 7DQ UK; 2 Perspectum Ltd, Oxford, UK; 3 Medical Physics, Guy’s and St Thomas’ NHS Foundation Trust, London, UK; 4 Biomedical Engineering Department, School of Biomedical Engineering and Imaging Sciences, King’s College London, London, UK; 5 Oxford Centre for Magnetic Resonance, Radcliffe Department of Medicine, University of Oxford, Oxford, UK; 6 Oxford NIHR Biomedical Research Centre, University of Oxford, Oxford, UK; 7 Translational Gastroenterology Unit, University of Oxford, Oxford, UK

**Keywords:** Liver, Iron, Magnetic resonance imaging, Thalassemia, Hemochromatosis

## Abstract

**Purpose:**

R2*, a measurement obtained using magnetic resonance imaging (MRI) can be used to estimate liver iron concentration (LIC). 3 T and 1.5 T scanners can be used but conversion of 3 T R2* to LIC is less well validated. In this study the aim was to compare 3 T-R2* LIC and 1.5 T-R2* LIC estimations to assess if they can be used interchangeably.

**Methods:**

Thirty participants were scanned at both 1.5 T and 3 T. R2* was measured at both field strengths. 3 T R2* and 1.5 R2* were compared using linear regression and were converted to LIC using different calibration curves. Pearson’s rho and Intraclass Correlation Coefficients (ICCs) were used to assess correlation and agreement between 1.5 and 3 T LIC. Bland Altman plots were used to assess bias and limits of agreement.

**Results:**

All 1.5 T and 3 T LIC comparisons gave Pearson’s rho of 0.99 (*p* < 0.001). ICC ranged from 0.83 (*p* = 0.005) to 0.96 (*p* < 0.001). Biases had magnitude of less than 0.2 mg/g dry weight.

**Conclusion:**

Agreement and bias between 3 and 1.5 T-R2* LIC depended on the method used for conversion. There were instances when the agreement was excellent and bias was small, indicating that potentially 3 T-R2* LIC can be used alongside or instead of 1.5 T-R2* LIC but care needs to be taken over the conversion methods selected.

**Trial registration number:**

Clinicaltrials.gov NCT03743272, 16 November 2018.

**Graphical abstract:**

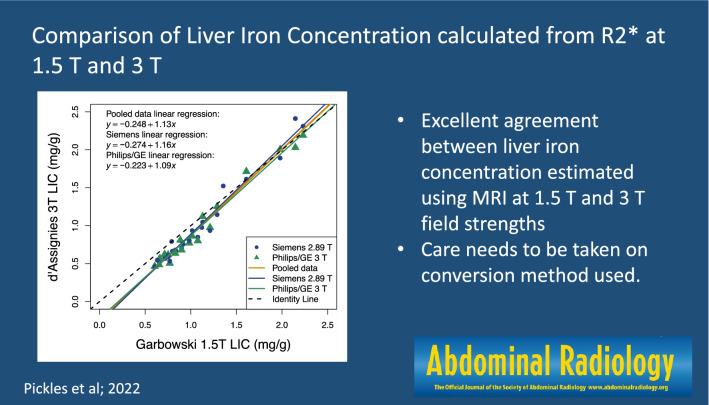

## Introduction

Elevation of liver iron concentration (LIC) occurs in patients with diseases such as hereditary haemochromatosis and fatty liver disease. LIC elevation can also result from having multiple blood transfusions, such as in patients with thalassemia. Increased LIC can lead to liver fibrosis, cirrhosis and cancer [1], so it is important to accurately diagnose and monitor LIC.

Multi-echo gradient echo sequences in MRI can be used to measure R2*, from which LIC can be estimated, using calibration curves from previous studies [2–5]. LIC estimated from 1.5 T R2* is widely used and is established as a method which is regarded as having reliable precision and accuracy for clinical practice [6].There are some, but currently fewer, methods available to convert R2* data acquired with 3 T scanners to LIC (either directly [7], or via a 3 T R2* to 1.5 T R2* conversion [8]). However, due to the limited number of studies at 3 T, the validity of these methods has not been verified to the same extent as the 1.5 T calibration curves. Given the increasingly widespread use of 3 T scanners, it is important that further studies are performed to assess the 3 T-R2* LIC conversion methods compared to 1.5 T-R2* LIC. One way to do this would be to assess agreement between 3 T-R2* LIC and 1.5 T-R2* LIC in the same subjects.

Assessing the agreement between 3 T-R2* LIC and 1.5 T-R2* LIC is important for several reasons. Firstly, given that 1.5 T-R2* LIC is used clinically, it is important that 3 T-R2* LIC is consistent with 1.5 T-R2* LIC so clinicians can confidently use 3 T-R2* LIC to diagnose iron overload.

Secondly, R2* to LIC conversion methods that work both at 3 T and 1.5 T would allow more flexibility at sites that would be able to use different scanners for the same indication. This is particularly the case in the treatment of diseases which involve patients receiving multiple transfusions such as thalassemia and certain cancers, which result in increasing iron content in the body. Sometimes chelation is administered to reduce the iron level. It is important therefore to monitor the impacts of transfusion and chelation on iron content in the body. Measuring LIC on an annual basis using MRI is the recommended method for monitoring the impact of chelation therapy [6, 9]. So that patients can be scanned on both 1.5 T and 3 T scanners during treatment, LIC calculated from 1.5 T scanners and 3 T scanners must be in close agreement. Additionally, if LIC can be measured at 3 T, then it would facilitate the conduct of clinical trials as sites would not be restricted to one type of scanner, but different sites could use 1.5 T or 3 T scanners.

The level of agreement required between 1.5 T LIC and 3 T LIC depends on the application. For categorising iron overload, the categories are quite broad (e.g. 3.2–7.0 mg/g dry weight (dw) is mild overload, 7.0–15.0 mg/g dw is moderate overload and > 15.0 mg/g dw is severe)[10]. Therefore, for diagnosis, a moderate amount of variability in LIC across field strengths is acceptable, e.g. ± 0.5 mg/g dw.

However, for monitoring treatment in the same patient across different scanners at both 1.5 T and 3 T, a closer agreement between 1.5 T and 3 T LIC may be required. For example, in a study of patients receiving the iron chelator Deferasirox, the change in least squares mean LIC was 1.95 mg/g dw over 52 weeks [11]. This works out at 0.45 mg/g dw every 12 weeks, so if monitoring of LIC was done on a 12-weekly basis then a variability no greater than 0.23 mg/g dw in results across field strengths may be required.

In terms of the methods available for calculating 3 T-R2* LIC, there is one calibration curve available to convert R2* to LIC at 3 T [7]. Another method to obtain 3 T-R2* LIC is to convert the R2* at 3 T to R2* at 1.5 T and then convert the 1.5 T R2* to LIC using the widely used 1.5 T calibration curves. The hypothesis is that 3 T-R2* LIC methods will give LIC which is in close enough agreement to 1.5 T-R2* LIC, to give clinicians and researchers the confidence to use 3 T-R2* LIC in diagnosis and monitoring of patients, both in the clinical setting and for research.

One way to test this hypothesis is to scan patients at both 3 T and 1.5 T and convert R2* to LIC at both field strengths and assess the agreement. The aim of this study was therefore to compare paired 1.5 T and 3 T R2* data that were converted to LIC, using a number of different LIC conversion methods. The agreement between the results at 1.5 T and 3 T was then assessed.

## Methods

### Patients

30 participants were recruited as part of the “Repeatability and Reproducibility of Multiparametric MRI” study (Clinicaltrials.gov NCT03743272). The inclusion criteria were: male and female subjects aged between 18 and 75 years, and ability to understand and sign a written informed consent form (ICF). A subset had previously been diagnosed with high liver fat or high iron. The trial had ethical approval (South-Central Oxford Research Ethics Committee C (Ref: 17/SC/0205)) and all participants gave written informed consent. Participant characteristics are given in Table 1.

**Table 1:** Participant characteristics

	Median (range)​	*N​*
*Gender*		
Male​		15​
Female​		15​
Age (years)​	36 (19–73)​	
BMI (kg/m^2^)​	25.4 (19.5–35.3)​	
Normal/Healthy Weight (18.5–24.9 kg/m^2^)​		14​
Overweight (25.0–29.9 kg/m^2^)​		10​
Obese (> 30.0 kg/m^2^)​		6​
Weight (kg)​	79.2 (47–102.3)​	
Height (cm)​	173 (147–192)​	
Reported healthy​		19​
Reported liver disease​		11​
Disease details (if available)​		
Fatty liver​		8​
Unhealthy (AIH, PSC, Wilson’s Disease)​		3​

### MRI protocol and post processing

Ten participants were scanned on one 1.5 T scanner and one 3 T scanner and twenty participants were scanned on one 1.5 T scanner and on two 3 T scanners. The resulting number of data points (1.5 T and 3 T paired data) was 50. All participants had fasted for 3–5 h before each scan and for each participant all scans were performed on the same day.

Three major scanner vendors were represented (Siemens, Philips and General Electric (GE)) and a variety of models were used. The scanner models are shown in Table 2, with the number of participants scanned on each. It should be noted that “3 T scanners” are not always exactly 3.00 T: Siemens 3 T scanners are actually 2.89 T, while Philips and GE 3 T scanners really are 3.0 T.

**Table 2:** Scanner vendor, model, field strength and number of scans per scanner

	Field strength ​	*N*​
*Siemens*		
Prisma​	3 T​	20​
Prisma_fit​	3 T​	10
Avanto_fit​	1.5 T​	10​
*Philips Medical Systems*​		
Achieva dStream​	3 T​	10​
Ingenia​	1.5 T​	10​
*GE Medical Systems*		
Discovery MR750​	3 T​	10​
Optima MR450w​	1.5 T​	10

On each scanner a multi-echo gradient echo sequence was used to acquire data for R2* mapping. The corresponding parameters are shown in Table 3. The acquisition was of a 2D slice and was obtained within a breath-hold of approximately 10 s. Parameters were different between field strengths to be appropriate for the expected range of R2*. At the same field strength, the parameters also varied slightly between scanners.

**Table 3:** Sequence parameters, range/approximation due to slight variation between scanners

Parameter	Siemens 1.5 T	Siemens 3 T	Philips 1.5 T	Philips 3 T	GE 1.5 T	GE 3 T
Field of View (mm x mm)	440 × 357		440 × 378		440 × 352	
Phase FOV (%)	81.25		85.9375		80	
Slice Thickness (mm)	3					
Number of Measurements	7	12	7	12	1	
Number of Slices	1		1		7	12
Slice gap	N/A		N/A		0	0
Parallel imaging technique	iPAT (twofold with 35 reference lines)		SENSE (twofold)		ARC 2.0	
Basic Matrix	128 × 104		142 × 140		128 × 128	
Reconstruction Matrix	256 × 208		256 × 256			
Reconstructed Voxel Size (mm x mm)	1.72 × 1.72					
Flip Angle	15	9	15	9	15	9
Number of Echoes	8					
First TE (ms)	2.38	1.23–1.33	2.37	1.18–1.32	1.816 –2.216	1.06 –1.536
Echo spacing (ms)	2.38	1.23	2.37–2.38	1.18–1.19	2.376–2.79	1.232
TR (ms)	~ 21	~ 11	~ 21	~ 11	~ 21	~ 11

At 1.5 T and 3 T the sequence involved acquiring, respectively, 7 and 12 repeated slices (at the same location). Having multiple repeats improves the signal to noise ratio (SNR) and allows sub-selection to avoid including data impacted by artifacts such as those due to motion. The algorithm used for processing the raw echoes first involves the generation of T2* maps for each repeat, then the automatic sub-selection of the most similar repeats (4 for 1.5 T and 7 for 3 T). This is followed by averaging of the raw echoes of that subset of repeats, and the generation of a T2* map from those averages.

The MAGO method was used to obtain T2* [12]. MAGO uses a multipeak fat spectrum and multipoint search approach to quantify both T2* and Proton Density Fat Fraction(PDFF). The MAGO technique forms part of LiverMultiscan v3 (Perspectum, Oxford, UK), a regulatory-cleared (FDA and CE) software. It provides a method for estimating PDFF and T2* without requiring a field map [12].

Once T2* maps were obtained trained analysts placed three circular ROIs, each 15 mm in diameter, in representative areas of the liver parenchyma, so as to avoid artifacts, large blood vessels and bile ducts. A median T2* of the pixels included in each ROI was calculated. The median T2* (units: ms) was then converted to R2* in s^−1^.

### 1.5 T LIC conversion

Several different 1.5 T calibration curves have been published. A previous study showed good agreement between a number of calibration curves [4], suggesting that it may be acceptable to use any one of them. In this study, Wood’s equation [2] and Garbowski’s equation [5] were used.

Wood’s equation is:

$$LIC\left(mg/g\right)=0.025 {R2}_{1.5 T}^{*}+0.202$$

Garbowski’s equation is

$$LIC\left(mg/g\right)=0.032 {R2}_{1.5 T}^{*}-0.14$$

### 3 T LIC conversion

Given that 3 T R2* and 1.5 T R2* are in theory strictly proportional to each other, one way of converting 3 T R2* to LIC is to firstly convert 3 T R2* to 1.5 T R2* and then use a 1.5 T-R2* LIC conversion curve to calculate the LIC, Alam [6] found the relationship between 1.5 T-R2* and 3 T-R2* to be

$${R2}_{3 T}^{*}=2.051 {R2}_{1.5 T}^{*}-25.4276$$

Linear regression equations between 1.5 T and 3 T R2* were found using data in this study and compared with the linear regression found by Alam.

Direct conversion from 3 T-R2* to LIC is also possible using d’Assignies’ calibration curve [7]. In their paper, d’Assignies’ et al. give two equations, one of which was for patients with LIC of < 7.26 mg/g dw. We chose this equation for our study as it was the more relevant to the LIC range for the population we examined. The d’Assignies equation is given in their paper in μmol/g dw, so this was converted to mg/g dw using the atomic weight of iron. The d’Assignies equation becomes:

$$LIC\left(mg/g\right)=0.0175 {R2}_{3 T}^{*}-0.536$$

### Statistical analysis

The relationship between 1.5 T and 3 T R2* was assessed using linear regression. The relationship between the 3 T R2*-LIC and 1.5 T-R2* LIC metrics was assessed using linear regression, and the correlation using Pearson’s rho. Agreement between the two measurements was assessed using the Intraclass Correlation Coefficient (ICC) and Bland Altman analysis (by calculating the bias and 95% limits of agreement (LoA)). The relationship and agreement was also assessed visually using scatter plots and Bland Altman plots with different colours and shapes chosen to distinguish between scanner types. All normality assumptions were assessed and validated using the Shapiro–Wilk test for normality.

## Results

**Fig. 1:**
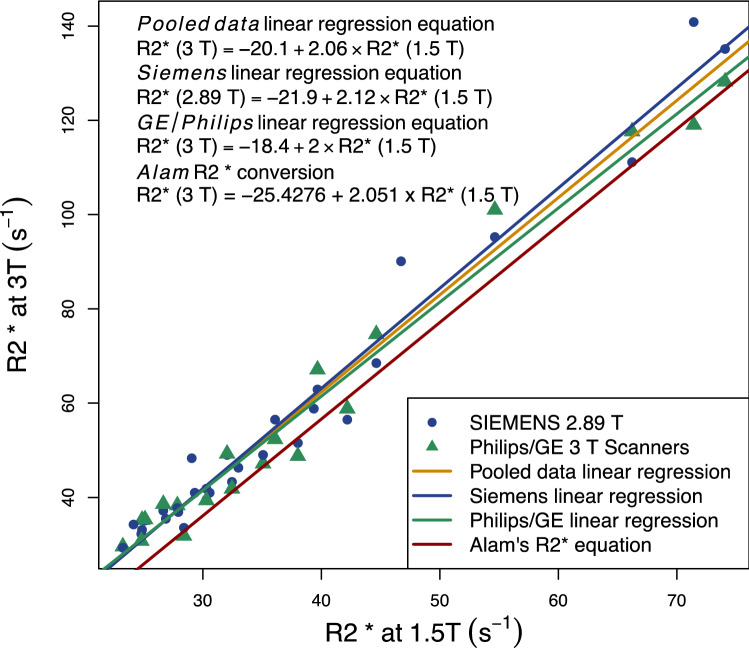
3 T R2* plotted against 1.5 T R2* with Alam's conversion line, and the linear regression lines using the data. Data point colour and symbol depends on whether the scanner was Siemens or Philip/GE. Three regression lines are plotted: for all data (pooled), Siemens data and Philips/GE data

The mean R2* at 1.5 T and 3 T were 37.5 s^−^^1^ (range: 23.4 s^−1^–74.1 s^−1^) and 57.1 s^−1^ (range: 29.3 s^−1^–140.8 s^−1^). The relationship between R2* at 3 T and 1.5 T, with fitted linear regression lines and equations, alongside Alam’s conversion, is shown in Fig. 1, with 3 T R2* being close to half of 1.5 T R2*. The linear regressions found from the data in this study are similar to Alam’s. There is a difference of less than 0.5% in the proportionality of the 1.5 T–3 T relationship between this study and Alam’s.

**Fig. 2:**
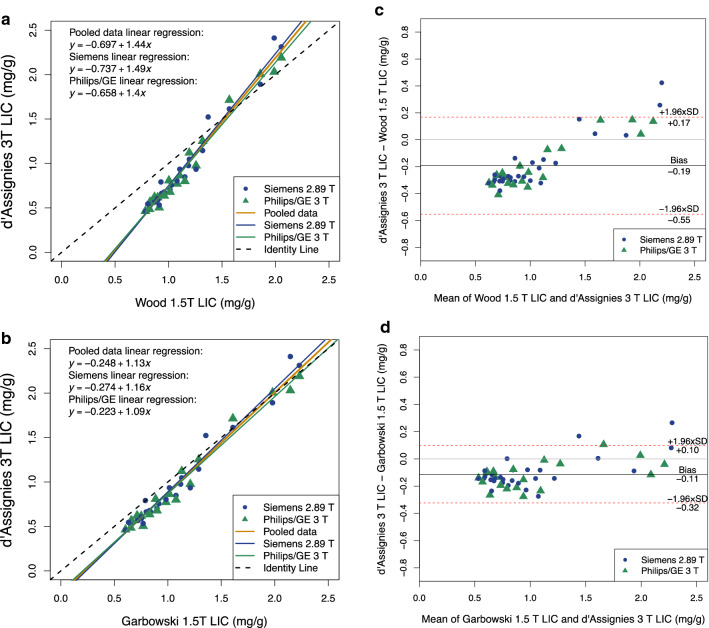
Scatter plots (**a**, **b**) and Bland Altman plots (**c**, **d**) comparing 1.5 T-R2* LIC and 3 T-R2* LIC using d’Assignies 3 T-R2* LIC conversion and **a, c**: Wood’s 1.5 T-R2* LIC conversion and **b**, **d**: Garbowski’s 1.5 T-R2* LIC conversion. Data point colour and symbol depends on whether the scanner was Siemens or Philip/GE. Three regression lines are plotted: for all data (pooled), Siemens data and Philips/GE data

Figure 2 shows the comparison of LIC from 1.5 T with LIC from 3 T using scatter plots and Bland–Altman plots. Pearson’s rho and ICC values are shown in Table 4. Bias and LoA are given in Table 5.

**Table 4:** Pearson's rho and Intraclass correlation coefficients for comparisons between 1.5 T-R2* LIC and 3 T-R2* LIC

	3 T Vendor	Number of samples	Pearson’s rho (95% CI)	Pearson’s rho *p* value	ICC (95% CI)	ICC *p* value
d’Assignies 3 T LIC compared with Wood 1.5 T LIC	All data	50	0.99 (0.97, 0.99)	*p* < 0.001	0.84 (0.28, 0.95)	0.004
	Siemens	30	0.99 (0.97, 0.99)	*p* < 0.001	0.83 (0.26, 0.94)	0.005
	Philips/GE	20	0.99 (0.97, 1.00)	*p* < 0.001	0.87 (0.28, 0.96)	0.005
d’Assignies 3 T LIC compared with Garbowski 1.5 T LIC	All data	50	0.99 (0.97, 0.99)	*p* < 0.001	0.95 (0.65, 0.99)	*p* < 0.001
	Siemens	30	0.99 (0.97, 0.99)	*p* < 0.001	0.95 (0.69, 0.98)	*p* < 0.001
	Philips/GE	20	0.99 (0.97, 1.00)	*p* < 0.001	0.96 (0.52, 0.99)	0.002

**Table 5:** Bland Altman statistics for 1.5 T-R2* LIC and 3 T-R2* LIC

	Number of samples	Bias (95% CI)	Lower LoA (95% CI)	Upper LoA (95% CI)
d’Assignies 3 T LIC and Wood 1.5 T LIC	50	− 0.19 (− 0.25, − 0.14)	− 0.55 (− 0.64, − 0.46)	0.17 (0.08, 0.26)
d’Assignies 3 T LIC compared with Garbowski 1.5 T LIC	50	− 0.11 (0.14, − 0.08)	− 0.32 (− 0.38, − 0.27)	0.10 (0.05, 0.15)

The scatter plots (Fig. 2) and Pearson’s rho (given in Table 4) show that 3 T-R2* LIC and 1.5 T-R2* LIC had strong correlation, regardless of the method used. However, the regression lines when Garbowski’s method for 1.5 T-R2* LIC was used were closer to the identity line compared to Wood’s 1.5 T-R2* LIC. The ICCs (Table 4) confirm that Garbowski’s 1.5 T-R2* LIC had greater agreement with d’Assignies’ 3 T- R2* LIC compared to Wood. Additionally, the confidence intervals for the ICCs were smaller.

The bias and limits of agreement depended on the 1.5 T-R2* to LIC method, as shown by the Bland Altman plots in Fig. 2, and Table 4, with smaller bias and tighter limits of agreement when Garbowski’s conversion was used. Bias was − 0.1 mg/g dw when Garbowski’s method was used, comparing to − 0.2 mg/g dw when Wood’s conversion was used.

Also of note, the Bland Altman plots, particularly for Wood, show a positive relationship between the bias and the magnitude of the paired values.

Splitting the data depending on whether the 3 T scanner was Siemens (2.89 T) or Philips/GE generally made little difference on the regression lines, correlation, and agreement, regardless of 1.5 T conversion method used.

## Discussion

It is important that iron levels are estimated sufficiently accurately so that clinically significant changes in iron can be detected. LIC estimated from 1.5 T R2* is widely used and is established as a method which is regarded as having sufficiently reliable precision and accuracy for clinical practice [6].

However, 3 T-R2* LIC is not as widely used and there are fewer studies to verify its accuracy. In this study, 3 T-R2* LIC was compared with 1.5 T-R2* LIC, to assess whether 3 T-R2* LIC can be used by clinicians and researchers with the same confidence that 1.5 T-R2* LIC is used. Additionally, comparing 3 T-R2* LIC with 1.5 T-R2* LIC enables the assessment of whether 1.5 T-R2* LIC and 3 T-R2* LIC can be used interchangeably, for example when a patient undergoing monitoring is scanned at both 1.5 T and 3 T or if a clinical trial is performed at both field strengths. 30 participants were scanned on multiple scanners to enable this comparison.

One method of converting to 3 T R2* to LIC could be by converting 3 T R2* to 1.5 T R2* and then using a 1.5 T-R2* LIC calibration curve, In this study 1.5 T and 3 T LIC had a linear relationship which was as expected, as R2* is in theory directly proportional to field strength. It was similar regardless of whether all the data were included or whether it split by vendor and was similar to the relationship found by Alam, with 3 T R2* being about half of 1.5 T R2*. This is also similar to the relationship found in other studies [13, 14]. One way to obtain similar LIC across two field strengths therefore could be to firstly to covert 3 T R2* to 1.5 T R2* and then convert 1.5 T R2* to LIC using the preferred 1.5 T LIC calibration curve.

A direct 3 T-R2* LIC calibration curve is also available. In this study, the LIC found using d’Assignies’ 3 T-R2* LIC calibration curve was compared with Wood’s 1.5 T-R2* LIC and Garbowski’s 1.5 T-R2* LIC. The results showed there was strong correlation and bias of less than 0.2 mg/g dw for both 1.5 T-R2* LIC conversion methods. However lower limits of agreement of − 0.55 mg/g dw and − 0.32 mg/g dw were found when using Wood and Garbowski’s conversion, respectively. One reason for the difference between 1.5 T-R2* LIC and 3 T-R2* LIC may be due to differences in patient populations between 1.5 T and 3 T. In d’Assignies population there were 49 patients with iron levels of 0-2 mg/g dw [7], whereas in both Wood’s [2] and Garbowski’s [5] studies there was only one patient with LIC below 2 mg/g dw. This therefore could impact the resulting calibration curve.

Garbowski’s 1.5 T-R2* LIC had greater agreement with d’Assignies 3 T-R2* LIC compared to Wood’s 1.5 T-R2* LIC. Additionally, a smaller bias and tighter limits of agreement were found. This is probably due to there being greater similarities between d’Assignies’ study and Garbowski’s study compared to Wood. For example, the quantification of biopsies in d’Assignies’ study and Garbowski’s study was in the Rennes laboratory, whereas in Wood’s study quantification was performed at Mayo Medical Laboratory. Differences in methods in processing and analysing biopsies can yield different results, and so the same laboratory being used for d’Assignies’ and Garbowski’s study may be one reason for the similar results. Additionally, similarity between sequence methods may be a factor. Both d’Assignies and Garbowski used multi-echo gradient echo techniques, whereas Wood’s study used a single-echo acquisition, repeated multiple times. Wood’s study also contained fewer biopsies (22) [2] compared to Garbowski (50) [5] and d’Assignies (76 for generating the calibration curve used in this paper, 104 in total in the study) [7].

The calibration curves may also differ due to the use of different fitting algorithms and software to produce the curves, as highlighted by Meloni [15]. Wood used a model which included noise as a variable offset, Garbowski used a truncation model for noise and d’Assignies used a noise subtraction algorithm which subtracted mean background noise [2, 5, 7]. It should be noted that the method of noise subtraction used by d’Assignies may not be appropriate, given the Rician nature of MR noise [16].

From the Bland Altman plots there appears to be an increasing positive difference between the 3 T-R2* LIC and 1.5-R2* LIC when both Wood and Garbowski’s methods are used, which may indicate that at higher iron levels the difference between 3 T-R2* LIC and 1.5 T-R2* LIC may be even greater. However, further verification of this is needed by acquiring data in patients with higher LIC (as in this study all participants had R2* equivalent to LIC below 2.5 mg/g dw, regardless of method used). It may be that using d’Assignies’ equation which included patients with LIC above 7.26 mg/g dw would improve the agreement between the methods at higher LIC.

Comparison was also made when data were split into Siemens 2.89 T and Philips/GE 3.0 T. However, there was very little difference in the linear regression equations, and the correlation and agreement, when compared to the pooled data results. This is perhaps to be expected when using d’Assignies for 3 T-R2* LIC, as that study included both Siemens and Philips 3 T scanners [7].

The improved agreement, bias and limits of agreement between d’Assignies and Garbowski compared to d’Assignies and Wood indicate that careful choice is required when choosing calibration curves. Given that factors such as the population, acquisition technique and laboratory analysis may all influence the resulting calibration curve, it is important that, if using both 1.5 T and 3 T for estimating LIC within the same hospital/centre, the calibration curves used have been acquired using as similar methodology as possible. However, it should be noted that the bias when using Wood was still below 0.23 mg/g dw and so it still may be clinically acceptable to use Wood for 1.5 T-R2* LIC and d’Assignies for 3 T-R2* LIC. It should also be noted that it is preferable to use 1.5 T R2* for LIC evaluation in patients with high iron overload, as at 3 T the fast signal decay can make R2* estimation challenging.

There were several limitations to our study. Firstly, no biopsy paired LIC was available, which is currently accepted as the ground truth for LIC. However, 1.5 T-R2* LIC is widely accepted and so it would be hard to ethically justify performing biopsies when 1.5 T-R2* LIC is available. Secondly, this study only involved participants with R2* equivalent to LIC below 2.5 mg/g dw (regardless of R2* to LIC conversion). Given that mild iron overload is considered to be 3. 2 mg/g dw – 7 mg/g dw [10] no participants had iron overload and so are not representative of the majority of people being imaged to establish LIC levels. Further studies are required on participants with higher LICs, but this study will provide a good foundation whilst other studies are awaited. Thirdly, the study only contained 30 participants. However, this was enough to establish correlations and agreements between 1.5 T-R2* LIC and 3 T-R2* LIC. Another limitation of this work is that all data were acquired and analysed using the same techniques. Using different acquisitions technique, and different software, could impact the results.

In conclusion, this study suggests that LIC estimated using R2* measured at 3 T may be similar to LIC measured using R2* at 1.5 T. However, care should be taken on which method is used to convert from R2* to LIC.

## Data Availability

Data available on request.
